# Supplementary material for: Hybrid Models and Biological Model Reduction with PyDSTool
Source: PLoS Comput Biol. 2012 Aug 9;8(8):e1002628. doi: 10.1371/journal.pcbi.1002628 (PMC3415397; doi:10.1371/journal.pcbi.1002628)
Supplement: Text S4 — Complete source code for the PyDSTool package (version 0.88.120504). Includes API documentation and help files linking to web pages. This file is identical to the current public release on Sourceforge.net. (ZIP) [file pcbi.1002628.s004.zip › PyDSTool/html/PyDSTool.Toolbox.optimizers.criterion.criteria.RelativeParametersCriterion-class.html]

xml version="1.0" encoding="ascii"?


PyDSTool.Toolbox.optimizers.criterion.criteria.RelativeParametersCriterion


| Home | Trees | Indices | Help | PyDSTool |
| --- | --- | --- | --- | --- |

Package PyDSTool ::
Package Toolbox ::
Package optimizers ::
Package criterion ::
Module criteria ::
Class RelativeParametersCriterion

[hide private]

[frames] | no frames]

# Class RelativeParametersCriterion

source code

```
object --+
         |
        RelativeParametersCriterion
```

---

The relative criterion stops the optimization when the relative error
of the parameters is below a certain level (xtol)


|  |  |
| --- | --- |
| Instance Methods | [hide private] |

|  |  |
| --- | --- |
| \_\_init\_\_(self, xtol, weight=None)  Initializes the criterion with an error fraction and the weight assigned for each parameter | source code |

|  |  |
| --- | --- |
| \_\_call\_\_(self, state, \*\*kwargs)  Computes the stopping criterion | source code |

**Inherited from `object`**:
`__delattr__`,
`__getattribute__`,
`__hash__`,
`__new__`,
`__reduce__`,
`__reduce_ex__`,
`__repr__`,
`__setattr__`,
`__str__`


|  |  |
| --- | --- |
| Properties | [hide private] |

**Inherited from `object`**:
`__class__`


|  |  |
| --- | --- |
| Method Details | [hide private] |

|  |  |
| --- | --- |
| \_\_init\_\_(self, xtol, weight=None)  *(Constructor)* | source code |

Initializes the criterion with an error fraction and the weight
assigned for each parameter

Overrides: object.\_\_init\_\_

  


| Home | Trees | Indices | Help | PyDSTool |
| --- | --- | --- | --- | --- |

|  |  |
| --- | --- |
| Generated by Epydoc 3.0.1 on Fri May 4 15:24:09 2012 | http://epydoc.sourceforge.net |
